# Supplementary material for: Socioeconomic roots of academic faculty
Source: Nat Hum Behav. 2022 Aug 29;6(12):1625–33. doi: 10.1038/s41562-022-01425-4 (PMC9755046; doi:10.1038/s41562-022-01425-4)
Supplement: Supplementary file 1 — Supplementary Tables 1–7 and Fig. 1. [file 41562_2022_1425_MOESM1_ESM.pdf]

nature portfolio

https://doi.org/10.1038/s41562-022-01425-4

Supplementary information

Socioeconomic roots of academic faculty

In the format provided by the authors and unedited

Socioeconomic Roots of Academic Faculty

Allison C. Morgan,[1,*] Nicholas LaBerge,[1,†] Daniel B. Larremore,[1,2,‡]
Mirta Galesic,[3,§] Jennie E. Brand,[4,¶] and Aaron Clauset[1,2,3,**]

[1] *Department of Computer Science, University of Colorado, Boulder, CO, USA*
[2] *BioFrontiers Institute, University of Colorado, Boulder, CO, USA*
[3] *Santa Fe Institute, Santa Fe, NM, USA*
[4] *University of California, Los Angeles, CA, USA*

Supplementary Information

A. Survey Details

Our survey was conducted over three years, from Summer 2017 to Fall 2020. The sample frame was assembled by drawing a set of PhD-granting departments from the lists maintained by the U.S. News & World Report for history and business, and the Computing Research Association for computer science, as in Ref. [1]. The sample frames for Anthropology, Biology, History, Physics/Astronomy, Psychology, and Sociology come from a comprehensive database of employment records for tenure-track faculty at U.S. PhD granting institutions that the Academic Analytics Research Center (AARC) provides. These data are based on a census of U.S. tenured and tenure-track faculty obtained under a Data Use Agreement with AARC, which scraped the data from public university websites and departmental faculty directories. (Requests for access to AARC data can be submitted at `https://aarcresearch.com/access-our-data`.) Then, email addresses listed in online public directories were collected for faculty in our sample frame using both automated methods and Mechanical Turk [1, 2].

Each individual received one email reminder. All participants were included in a drawing for a cash lottery. Amounts varied by survey wave: one $1,000 payment for Computer Science, $500 for Business and History faculty, $500 for Anthropology, Physics / Astronomy, Psychology, and Sociology faculty, and $250 for Biology faculty. To reduce the burden on participants, our survey was divided into two parts. The first part took 1-2 minutes to complete and asked the most important questions of interest for our study, including participants' year of birth, childhood ZIP code, and information on their parents' education and employment. After completing the first part, participants were told that they can continue to the second part, which asked about parental support for their careers. Our study was approved by the University of Colorado Boulder Institutional Review Board.

In total, 8,009 faculty responded to our survey (out of 46 692 surveyed; 17.2%). Of those, 7,204 faculty provided information on a parent's level of highest education (89.9% of respondents) and 4,807 provided the ZIP code in which they grew up (60.0%). If a ZIP code was not provided, an open text box to specify city, state, and / or country was available. Overall, our response rate mirrors other online surveys with email invitations conducted in the context of academia [3, 4]. Faculty that provided either parents' education or ZIP code for our analysis are generally representative with respect to their populations. Women responded at slightly higher rates than expected (Supplementary Table I), which may imply a slight upward bias in our results in parents' education. In our sample, women are somewhat more likely to come from highly educated families (one of their parents holds a PhD: 24.8% versus 20.8%; $z = 3.9$, $N = 7,145$, $p < 0.001$). Respondents came from 2,494 unique ZIP codes across the U.S. (Supplementary Figure 1). Most ZIP codes were represented by only one respondent (76.9%). Questions about degree of parental support were asked towards the end of the survey (85.9% of respondents). Those respondents had similar demographics to those who provided parental education information (Supplementary Table I), but skewed towards being from slightly less prestigious institutions in History (56.6 versus 51.8, Kolmogorov-Smirnov, $D = 0.1$, $p = 0.032$), and slightly more in Sociology (38.8 versus 40.0, Kolmogorov-Smirnov, $D = 0.1$, $p = 0.039$).

Survey Questions

- "In what year were you born?" Drop down of years from 1916 to 1996.
- "During the first 18 years of your life, did your family rent the home in which you lived, or did your family own it (even if supported by a mortgage)?" Options were "We rented a home during all or most of the first 18 years of my life," "We rented and owned a home about equally often," "We owned a home during all or most of the first 18 years of my life," or "Don't know"
- "Where did you live during the first 18 years of your life? If you lived in the U.S., please let us know in which ZIP code you live the longest." Open text box.

* allison.morgan@colorado.edu
† nicholas.laberge@colorado.edu
‡ daniel.larremore@colorado.edu
§ galesic@santafe.edu
¶ brand@soc.ucla.edu
** aaron.clauset@colorado.edu

Field		Men	Women	Nonbinary	Undisclosed	Assistant	Associate	Full	Prestige	N
**Anthropology**	Respondents	48.7	50.1	0.6	0.6	17.4	30.6	52.1	26.5 (21.5)	355
	Population	50.8	49.2	–	–	21.1	32.1	46.7	22.6 (19.4)	1994
p-value									* 0.037	
**Biology**	Respondents	65.1	34.3	0.1	0.4	12.7	29.7	57.7	83.3 (61.4)	1549
	Population	70.1	29.9	–	–	23.1	27.6	49.3	83.2 (64.5)	10145
p-value		* < 0.001				* < 0.001				
**Business**	Respondents	66.7	32.4	0.1	0.8	30.8	25.7	43.5	42.4 (27.5)	1293
	Population	76.5	23.5	–	–	30.4	27.9	41.7	43.4 (28.7)	9573
p-value		* < 0.001								
**Computer Science**	Respondents	78.8	20.3	0.1	0.8	33.6	22.5	43.9	65.4 (47.2)	999
	Population	85.5	14.5	–	–	22.9	26.8	50.3	65.7 (48.5)	5792
p-value		* < 0.001				* < 0.001				
**History**	Respondents	53.0	46.5	0.2	0.2	16.8	39.3	43.9	55.8 (39.0)	992
	Population	62.7	37.3	–	–	16.4	38.3	45.3	51.9 (38.2)	4336
p-value		* < 0.001								
**Physics/Astronomy**	Respondents	79.3	19.6	0.2	1.0	17.5	18.6	63.8	52.4 (39.6)	937
	Population	85.7	14.3	–	–	18.1	19.7	62.2	51.5 (41.7)	5874
p-value									* 0.037	
**Psychology**	Respondents	46.5	53.1	0.2	0.2	20.7	30.4	48.9	83.8 (59.9)	982
	Population	54.4	45.6	–	–	23.1	28.7	48.2	88.2 (60.5)	6507
p-value		* 0.043							* 0.018	
**Sociology**	Respondents	45.6	53.7	0.2	0.4	17.5	31.9	50.6	38.7 (25.9)	447
	Population	50.0	50.0	–	–	22.1	29.7	48.2	40.0 (27.3)	2471
p-value										

Supplementary Table I. Demographic attributes of faculty in each discipline who provided either of the most important variables to this study. We report proportions for gender and faculty rank and means and standard deviations (in parentheses) for departmental prestige. Statistical significance was calculated via χ^2 two-tailed tests for gender and rank, and Kolmogorov-Smirnov two-tailed tests for prestige.

- "Now please think of your parents or legal guardians during the first 18 years of your life and answer the following questions about them. If you grew up with just one parent or legal guardian, please select 'Not applicable' for 'Parent 2'."
  - "What are their genders?" Options were "Male," "Female," or "Other identity."
  - "What is their highest level of education?" Options were "Elementary: 0-4 years," "Elementary: 5-8 years," "High school: 1-3 years," "High school: 4 years," "College: 1-3 years," "College: 4 or more years," "Master's or professional degree," "Doctoral degree," "Don't know," or "Not applicable / Rather not say."
  - "What best describes their employment status during all or most of the first 18 years of your life?" Options were "Employed," "Not employed: stay-at-home parent," "Not employed: could not find job," "Not employed: other reason (e.g. retired, illness, ...)," "Don't know or something else," or "Not applicable / Rather not say."

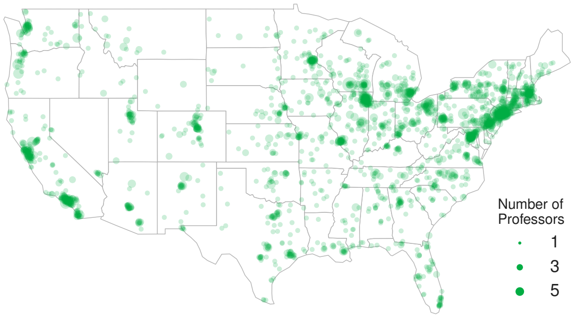

Supplementary Figure 1. Geographic plot of respondents by childhood ZIP code. Dot size scales with the number of faculty responses. Boundary and shape files for U.S. states and ZIP codes via Refs. [5, 6].

- "What is your gender?" Options were "Male," "Female," "Other identity," or "Prefer not to say."
- "What is your race or origin? Please select one or more responses." Options were "White," "Hispanic, Latino, or Spanish origin," "Black or African American," "Asian," "American Indian or Alaska Native," "Native Hawaiian or other Pacific Islander," "Some other race or origin," or "Prefer not to say."
- "Please rate support and encouragement you received for your academic career from people below, on a scale from 1 (none at all) to 5 (a lot). If

some of those people were not present in your life, please choose Not applicable." Category analyzed was "your parents" with options 1–5 and "Not Applicable."

B. Matching to Census, IRS, and NSF Data

Comparing the education and income of the parents of faculty with the overall United States population involves linking responses to publicly available education and income data. Responses were matched either by the year faculty were born or the year they began their first tenure-track job, to the appropriate year of data release.

For example, the National Science Foundation's Survey of Earned Doctorates (NSF SED) provides information on the educational attainment of the parents of doctoral recipients every five years from 1993 to 2018 (Supplementary Table IV). Survey responses were matched to the closest available data. In many cases (32.6% of responses) this was 1993, the earliest year available from the NSF.

Data on the urban or rural classifications of ZIP codes were obtained from the decennial U.S. Census presented by the Integrated Public Use Microdata Series (IPUMS) National Historical Geographic Information System (NHGIS) [8] (Supplementary Table V). Responses were linked by the closest year to when faculty were 21 years old, to represent the ZIP code's characteristics close to when the respondent was growing up. The majority of responses (67.9%) were matched to the earliest data release (1990).

The American Community Survey (ACS) provides estimates of the highest educational level attained by all adults (25 years or older) in the U.S. [9] (Supplementary Table VI). This data is available on a yearly basis starting in 1993 [10]. We link this data to responses by the birth year of faculty to compare the parents of faculty with the U.S. adult population at the time of their birth. Almost all responses are linked to the earliest release available.

Lastly, it is generally difficult to estimate income distributions for geographic locales, especially without a direct connection to census tract. Our approach using ZIP codes is common, in part because people can recall their ZIP code more readily than street address or census tract. The U.S. Internal Revenue Service (Supplementary Table VII) reports the total income and number of returns for each ZIP code in the U.S. from 1998 to 2018. Responses were linked by the closest year to when faculty were 21 years old, to approximate the wealth of a ZIP code during the person's childhood. Most responses (79.0%) were linked to the earliest release since the average year the faculty we surveyed were born was 1967. The IRS provides earlier data on the county level (from 1989 on; [12]), and state levels (from 1954 on; [13]). Because ZIP codes are based on the mail distribution needs of USPS, they only roughly correspond to other geographic locales [14]. For example, they change shape over time, and can span states or counties. Altogether, this means linking ZIP codes to historical income on the county and state level is difficult and requires the boundaries of ZIP codes and counties, and population estimates for each over time.

C. Comparing Survey Household Estimates with Population Estimates of Education

Assuming a two-parent household where each parent has an equal chance of having a PhD, and the percentage of U.S. adults without a Ph.D is 99.1% (Table 1), we estimate the percentage of children who have at least one PhD parent is roughly 1.8% (i.e., $1 - (0.991 \cdot 0.991) = 0.018$). This value divided by the proportion of faculty with at least one PhD parent returns a lower bound of 12 (0.222/0.018). Considering a single parent household, the upper bound becomes 25 (0.222/0.009; denominator is the percentage of adults with a PhD).

Obtaining precise bounds on this rate is difficult, as it depends on family structure and the strength of educational homogamy [15], and must account for the generally declining fertility by education level [16]. Based on current estimates of these factors, along with our data's overestimate of the U.S. adult population's PhD attainment rates, we expect the true value to be in the upper end of this range.

Unlike the U.S. Census data, the NSF SED reports the highest educational attainment of either parent, consistent with our results. Surveyed faculty are 1.9 times more likely as PhD recipients to have a parent who holds a PhD (0.222/0.188).

D. Relationships between SES, Age, and Geographic Distance

A scholar's search for a faculty job may be influenced by family SES, either by shaping how long they are willing to search for scarce tenure-track employment, or by geographically limiting which institutions they can apply to for jobs. Faculty who reported at least one of their parents holds a PhD are slightly younger at the start of the tenure-track job than those faculty whose parents do not hold a PhD (33.1 versus 33.6 years old; $t = -3.0$, $N = 6951$, $p = 0.002$). They traveled about the same distance from home though (873 versus 835 miles; Kolmogorov-Smirnov, $D = 0.0$, $N = 7209$, $p = 0.997$).

[1] Aaron Clauset, Samuel Arbesman, and Daniel B Larremore, "Systematic inequality and hierarchy in faculty hiring networks," Science Advances **1**, e1400005 (2015).

	Elementary	Some HS	HS	Some College	College	Masters	PhD
Anthropology Women Professors	0.6	1.7	16.9	5.6	18.5	32.0	24.7
Anthropology Men Professors	1.2	2.9	13.3	8.7	20.2	31.8	22.0
Biology Women Professors	1.7	2.6	9.7	12.5	18.9	30.5	24.0
Biology Men Professors	3.9	3.7	16.7	11.2	19.8	23.8	20.9
Business Women Professors	0.3	2.4	11.9	7.6	26.5	32.3	18.9
Business Men Professors	3.2	3.7	15.8	8.8	23.0	29.9	15.6
CS Women Professors	1.5	1.5	11.3	4.9	28.1	21.7	31.0
CS Men Professors	3.7	3.9	10.7	9.8	19.8	27.4	24.7
History Women Professors	1.7	1.2	7.9	5.7	15.3	37.3	30.9
History Men Professors	1.5	1.5	12.8	10.7	18.5	31.9	23.1
Physics/Astronomy Women Professors	3.3	3.8	7.7	12.0	13.1	31.1	29.0
Physics/Astronomy Men Professors	4.3	4.2	13.1	9.4	19.6	26.3	23.1
Psychology Women Professors	0.8	1.7	17.1	8.8	17.1	31.2	23.3
Psychology Men Professors	2.6	2.6	17.4	11.2	17.1	31.2	17.8
Sociology Women Professors	1.7	2.1	17.1	5.4	17.5	36.7	19.6
Sociology Men Professors	2.0	3.4	17.6	8.8	16.7	34.3	17.2

Supplementary Table II. Percentages of faculty by gender and discipline with their parents' highest held degree.

	Elementary	Some HS	HS	Some College	College	Masters	PhD
White Women Professors	0.9	1.7	12.1	7.3	19.5	32.9	25.5
Black Women Professors	4.9	–	17.5	14.6	11.7	36.9	14.6
Hispanic Women Professors	2.3	2.3	13.7	15.3	15.3	31.3	19.8
Asian Women Professors	2.4	4.4	7.3	12.5	19.4	30.2	23.8
American Indian / Native Women Professors	–	5.3	21.1	10.5	10.5	36.8	15.8
White Men Professors	2.4	2.5	15.2	9.6	19.2	29.1	22.0
Black Men Professors	7.4	8.4	15.8	12.6	9.5	26.3	20.0
Hispanic Men Professors	6.9	6.4	17.0	9.6	18.1	27.1	14.9
Asian Men Professors	7.3	8.4	7.5	12.4	25.3	23.4	15.7
American Indian / Native Men Professors	–	3.4	17.2	10.3	20.7	37.9	10.3

Supplementary Table III. Percentages of faculty by race / ethnicity and gender with their parents' highest held degree. Dashes denote zero respondents.

Year of NSF SED	Avg.	Min.	Max.	N
1993	1985	1928	1995	2326
1998	1998	1996	2000	868
2003	2003	2001	2005	985
2008	2008	2006	2010	985
2013	2013	2011	2015	1310
2018	2017	2016	2020	647

Supplementary Table IV. Number of responses (N) linked to each release of NSF SED [7]. NSF SED contains the educational attainment of the parents of doctoral recipients. The average, minimum, and maximum tenure track start year across linked responses.

Year of US Census	Avg.	Min.	Max.	N
1990	1960	1916	1974	4985
2000	1979	1975	1984	1978
2010	1987	1985	1999	379

Supplementary Table V. Number of responses (N) linked to each release of the U.S. Census [8]. The U.S. Census contains the percentage of the urban and rural population per ZIP code. The average, minimum, and maximum year faculty were born across linked responses.

[2] Allison C Morgan, Samuel F Way, and Aaron Clauset, "Automatically assembling a full census of an academic field," PloS ONE **13** (2018).

[3] Jennifer Dykema, John Stevenson, Lisa Klein, Yujin Kim, and Brendan Day, "Effects of E-Mailed Versus Mailed Invitations and Incentives on Response Rates, Data Quality, and Costs in a Web Survey of University Faculty," Social Science Computer Review **31**, 359–370 (2013).

[4] Morgan M. Millar and Don A. Dillman, "Improving Response to Web and Mixed-Mode Surveys," Public Opinion Quarterly **75**, 249–269 (2011).

[5] U.S. Census Bureau, "Cartographic Boundary Files - Shapefile, ZIP Code Tabulation Areas (zctas)," U.S. Department of Commerce (2018).

Year of ACS	Avg.	Min.	Max.	N
1993	1967	1916	1992	7338
1996	1998	1996	1999	4

Supplementary Table VI. Number of responses (N) linked to the American Community Survey [9, 10]. ACS reports the educational attainment of adults (25 years and older) in the United States. The average, minimum, and maximum year faculty were born across linked responses.

Year of IRS	Avg.	Min.	Max.	N
1998	1963	1916	1978	5802
2001	1980	1979	1980	394
2002	1981	1981	1982	422
2004	1983	1983	1983	177
2005	1984	1984	1984	168
2006	1985	1985	1985	118
2007	1986	1986	1986	111
2008	1987	1987	1987	61
2009	1988	1988	1988	44
2010	1989	1989	1989	25
2011	1990	1990	1990	11
2012	1991	1991	1991	4
2013	1992	1992	1992	1
2017	1996	1996	1996	2
2019	1999	1999	1999	2

Supplementary Table VII. Number of responses (N) linked to IRS data releases [11]. The IRS reports the total income and number of returns reported per ZIP code. The average, minimum, and maximum year faculty were born across linked responses.

[6] Natural Earth Data, "1:10m Cultural Vectors, Admin 1 - States, Provinces," (2016).

[7] National Science Foundation, "Doctorate Recipients from US Universities: 2018," National Center for Science and Engineering Statistics (2019).

[8] Steven Manson, Jonathan Schroeder, David Van Riper, Tracy Kugler, and Steven Ruggles, "IPUMS National Historical Geographic Information System," (2020).

[9] U.S. Census Bureau, "Table 3. Detailed Years of School Completed by People 25 Years and Over by Sex, Age Groups, Race and Hispanic Origin: 2010," U.S. Department of Commerce (2010).

[10] U.S. Census Bureau, "Table 4. Years of School Completed By Persons 25 Years Old and Over, By Race, For The United States, By Regions," U.S. Department of Commerce (1967).

[11] Internal Revenue Service, "SOI Tax Stats-Individual Income Tax Statistics-Zip Code Data (SOI)," (2019).

[12] Internal Revenue Service, "SOI Tax Stats-County Data," (2020).

[13] Internal Revenue Service, "SOI Tax Stats Archive-1954 to 1999 Individual Income Tax Return Reports," (1999).

[14] Tony H Grubesic, "Zip codes and spatial analysis: Problems and prospects," Socio-economic Planning Sciences **42**, 129–149 (2008).

[15] Christine R Schwartz and Robert D Mare, "Trends in educational assortative marriage from 1940 to 2003," Demography **42**, 621–646 (2005).

[16] Øystein Kravdal and Ronald R Rindfuss, "Changing relationships between education and fertility: A study of women and men born 1940 to 1964," American Sociological Review **73**, 854–873 (2008).
