# Supplementary material for: Socioeconomic roots of academic faculty
Source: Nat Hum Behav. 2022 Aug 29;6(12):1625–33. doi: 10.1038/s41562-022-01425-4 (PMC9755046; doi:10.1038/s41562-022-01425-4)
Supplement: Supplementary file 2 — Reporting Summary. [file 41562_2022_1425_MOESM2_ESM.pdf]

nature portfolio

Corresponding author(s): Allison Morgan

Last updated by author(s): Jun 8, 2022

Reporting Summary

Nature Portfolio wishes to improve the reproducibility of the work that we publish. This form provides structure for consistency and transparency in reporting. For further information on Nature Portfolio policies, see our Editorial Policies and the Editorial Policy Checklist.

Statistics

For all statistical analyses, confirm that the following items are present in the figure legend, table legend, main text, or Methods section.

n/a	Confirmed	
☐	☒	The exact sample size (*n*) for each experimental group/condition, given as a discrete number and unit of measurement
☒	☐	A statement on whether measurements were taken from distinct samples or whether the same sample was measured repeatedly
☐	☒	The statistical test(s) used AND whether they are one- or two-sided<br>*Only common tests should be described solely by name; describe more complex techniques in the Methods section.*
☐	☒	A description of all covariates tested
☒	☐	A description of any assumptions or corrections, such as tests of normality and adjustment for multiple comparisons
☐	☒	A full description of the statistical parameters including central tendency (e.g. means) or other basic estimates (e.g. regression coefficient) AND variation (e.g. standard deviation) or associated estimates of uncertainty (e.g. confidence intervals)
☐	☒	For null hypothesis testing, the test statistic (e.g. *F*, *t*, *r*) with confidence intervals, effect sizes, degrees of freedom and *P* value noted<br>*Give P values as exact values whenever suitable.*
☒	☐	For Bayesian analysis, information on the choice of priors and Markov chain Monte Carlo settings
☒	☐	For hierarchical and complex designs, identification of the appropriate level for tests and full reporting of outcomes
☒	☐	Estimates of effect sizes (e.g. Cohen's *d*, Pearson's *r*), indicating how they were calculated

Our web collection on statistics for biologists contains articles on many of the points above.

Software and code

Policy information about availability of computer code

Data collection	No software was used for data collection.
Data analysis	Publicly available Python (3.9.1) libraries for statistics and visualization: matplotlib (3.3.3), numpy (1.19.5), pandas (1.2.0), scipy (1.6.0), seaborn (0.11.1), statsmodels (0.12.1), and geopandas (0.8.2). All analysis code available at: https://github.com/allisonmorgan/faculty_ses

For manuscripts utilizing custom algorithms or software that are central to the research but not yet described in published literature, software must be made available to editors and reviewers. We strongly encourage code deposition in a community repository (e.g. GitHub). See the Nature Portfolio guidelines for submitting code & software for further information.

Data

Policy information about availability of data

All manuscripts must include a data availability statement. This statement should provide the following information, where applicable:
- Accession codes, unique identifiers, or web links for publicly available datasets
- A description of any restrictions on data availability
- For clinical datasets or third party data, please ensure that the statement adheres to our policy

Given the sensitive nature of the information provided by respondents to the survey, the underlying data cannot be de-identified in a way that would protect respondents’ privacy while also preserving the data’s utility for reanalysis or reuse. For these reasons, data can only be made available under an appropriate IRB-approved data sharing agreement.

Field-specific reporting

Please select the one below that is the best fit for your research. If you are not sure, read the appropriate sections before making your selection.

☐ Life sciences ☒ Behavioural & social sciences ☐ Ecological, evolutionary & environmental sciences

For a reference copy of the document with all sections, see nature.com/documents/nr-reporting-summary-flat.pdf

Behavioural & social sciences study design

All studies must disclose on these points even when the disclosure is negative.

Study description

We conducted a survey of professors in PhD-granting departments in the U.S. across STEM, social science, and the humanities, and provide a quantitative description.

Research sample

In total, 8,009 faculty responded to our survey (out of 46,692 surveyed; 17.2%). Of those, 7,204 faculty provided information on a parent's level of highest education (89.9% of respondents) and 4,807 provided the ZIP code in which they grew up (60.0%). Faculty that provided either parents' education or zip code for our analysis are generally representative with respect to their populations. Women responded at slightly higher rates than expected, which may imply a slight upward bias in our results in parents' education. In our sample, women are somewhat more likely to come from highly educated families. Respondents came from 2,494 unique ZIP codes across the U.S. Most ZIP codes were represented by only one respondent (76.9%). Questions about degree of parental support were asked towards the end of the survey (85.9% of respondents). Those respondents had similar demographics to those who provided parental education information, but skewed towards being from slightly less prestigious institutions in History, and slightly more in Sociology. More detail provided in Supplementary Information.

Sampling strategy

The sample frame was assembled by drawing a set of PhD-granting departments from the lists maintained by the U.S. News & World Report for history and business, and the Computing Research Association for computer science. The sample frames for Anthropology, Biology, History, Physics / Astronomy, Psychology, and Sociology are based on a comprehensive database of employment records for tenure-track faculty at U.S. PhD granting institutions that Academic Analytics–the academic consultancy that collected and maintains these faculty records provided for this research. Then, email addresses listed in online public directories were collected for faculty in our sample frame using both automated methods and Mechanical Turk.

Data collection

Relevant survey questions were as follows:

* ``In what year were you born?'' Drop down of years from 1916 to 1996.
* ``During the first 18 years of your life, did your family rent the home in which you lived, or did your family own it (even if supported by a mortgage)?'' Options were ``We rented a home during all or most of the first 18 years of my life,'' ``We rented and owned a home about equally often,'' ``We owned a home during all or most of the first 18 years of my life,'' or ``Don't know''
* ``Where did you live during the first 18 years of your life? If you lived in the U.S., please let us know in which ZIP code you live the longest.'' Open text box.

* ``Now please think of your parents or legal guardians during the first 18 years of your life and answer the following questions about them. If you grew up with just one parent or legal guardian, please select `Not applicable' for `Parent 2'.''
- ``What are their genders?'' Options were ``Male,'' ``Female,'' or ``Other identity.''
- ``What is their highest level of education?'' Options were ``Elementary: 0-4 years,'' ``Elementary: 5-8 years,'' ``High school: 1-3 years,'' ``High school: 4 years,'' ``College: 1-3 years,'' ``College: 4 or more years,'' ``Master's or professional degree,'' ``Doctoral degree,'' ``Don't know,'' or ``Not applicable / Rather not say.''
- ``What best describes their employment status during all or most of the first 18 years of your life?'' Options were ``Employed,'' ``Not employed: stay-at-home parent,'' ``Not employed: could not find job,'' ``Not employed: other reason (e.g. retired, illness, ...),'' ``Don't know or something else,'' or ``Not applicable / Rather not say.''

* ``What is your gender?'' Options were ``Male,'' ``Female,'' ``Other identity,'' or ``Prefer not to say.''
* ``What is your race or origin? Please select one or more responses.'' Options were ``White,'' ``Hispanic, Latino, or Spanish origin,'' ``Black or African American,'' ``Asian,'' ``American Indian or Alaska Native,'' ``Native Hawaiian or other Pacific Islander,'' ``Some other race or origin,'' or ``Prefer not to say.''
* ``Please rate support and encouragement you received for your academic career from people below, on a scale from 1 (none at all) to 5 (a lot). If some of those people were not present in your life, please choose Not applicable.'' Category analyzed was ``your parents'' with options 1--5 and ``Not Applicable.''

Survey was administered via emails, and recorded over the web. No blinding in response to experimental conditions were performed.

Timing

Our survey was conducted over three years, from Summer 2017 to Fall 2020.

Data exclusions

Any survey respondents who could not be identified from our frame were dropped. Otherwise no responses were excluded.

Non-participation

In total, 8,009 faculty responded to our survey (out of 46,692 surveyed; 17.2%). Of those, 7,204 faculty provided information on a parent's level of highest education (89.9% of respondents) and 4,807 provided the ZIP code in which they grew up (60.0%).

Randomization

All survey respondents received the same instruments. Our research questions did not require randomization.

Reporting for specific materials, systems and methods

We require information from authors about some types of materials, experimental systems and methods used in many studies. Here, indicate whether each material, system or method listed is relevant to your study. If you are not sure if a list item applies to your research, read the appropriate section before selecting a response.

Materials & experimental systems

n/a	Involved in the study
☒	☐ Antibodies
☒	☐ Eukaryotic cell lines
☒	☐ Palaeontology and archaeology
☒	☐ Animals and other organisms
☐	☒ Human research participants
☒	☐ Clinical data
☒	☐ Dual use research of concern

Methods

n/a	Involved in the study
☒	☐ ChIP-seq
☒	☐ Flow cytometry
☒	☐ MRI-based neuroimaging

Human research participants

Policy information about studies involving human research participants

Population characteristics	In total, 7,204 faculty provided information on a parent's level of highest education (15.4% of survey frame) and 4,807 provided the U.S. ZIP code in which they grew up (10.3%). Across the eight disciplines surveyed, women represented between 19.6% (Physics / Astronomy) and 53.7% (Sociology) of responses. Average year of birth of respondents was 1967.
Recruitment	Each individual received one email reminder. All participants were included in a drawing for a cash lottery. Amounts varied by survey wave: one $1,000 payment for Computer Science, $500 for Business and History faculty, $500 for Anthropology, Physics / Astronomy, Psychology, and Sociology faculty, and $250 for Biology faculty. To reduce the burden on participants, our survey was divided into two parts. The first part took 1-2 minutes to complete and asked the most important questions of interest for our study, including participants' year of birth, childhood ZIP code, and information on their parents' education and employment. After completing the first part, participants were told that they can continue to the second part, which asked about parental support for their careers.
Ethics oversight	University of Colorado Boulder IRB (Protocol #16-0441)

Note that full information on the approval of the study protocol must also be provided in the manuscript.
